# Relationship between Brain Metabolic Disorders and Cognitive Impairment: LDL Receptor Defect

**DOI:** 10.3390/ijms23158384

**Published:** 2022-07-29

**Authors:** Dong-Yong Hong, Dong-Hun Lee, Ji-Young Lee, Eun-Chae Lee, Sang-Won Park, Man-Ryul Lee, Jae-Sang Oh

**Affiliations:** 1 Department of Neurosurgery, College of Medicine, Soonchunhyang University, Cheonan Hospital, Cheonan 31151, Korea; dydehdghd@gmail.com (D.-Y.H.); madeby58@gmail.com (D.-H.L.); applesori82@gmail.com (J.-Y.L.); lec9589@gmail.com (E.-C.L.); ppphilio3@gmail.com (S.-W.P.); 2 Soonchunhyang Institute of Medi-Bio Science (SIMS), Soon Chun Hyang University, Cheonan 31151, Korea

**Keywords:** cholesterol metabolism, *LDLr*, insulin receptor, *SREBP*, blood–brain-barrier (BBB) breakdown, neuroinflammation, ER stress, mitochondria, apoptosis, lectin-like oxidized LDL receptor-1 (*LOX-1*)

## Abstract

The low-density-lipoprotein receptor (*LDLr*) removes low-density lipoprotein (LDL), an endovascular transporter that carries cholesterol from the bloodstream to peripheral tissues. The maintenance of cholesterol content in the brain, which is important to protect brain function, is affected by *LDLr*. *LDLr* co-localizes with the insulin receptor and complements the internalization of LDL. In *LDLr* deficiency, LDL blood levels and insulin resistance increase, leading to abnormal cholesterol control and cognitive deficits in atherosclerosis. Defects in brain cholesterol metabolism lead to neuroinflammation and blood–brain-barrier (BBB) degradation. Moreover, interactions between endoplasmic reticulum stress (ER stress) and mitochondria are induced by ox-LDL accumulation, apolipoprotein E (*ApoE*) regulates the levels of amyloid beta (Aβ) in the brain, and hypoxia is induced by apoptosis induced by the *LDLr* defect. This review summarizes the association between neurodegenerative brain disease and typical cognitive deficits.

## 1. Introduction

Low-density-lipoprotein receptor (*LDLr*) is involved in the regulation of blood cholesterol. *LDLr* internalizes cholesterol-containing LDL ligands, and insulin receptor (IR) maintains glycemic homeostasis. Although the exact mechanism is not completely clear, diabetes is associated with dyslipidemia. Diabetes is also characterized by abnormalities ranging from IR to absolute deficiency of insulin (type 1 diabetes) or abnormalities in the receptor protein itself post-transcription/translation/translation (type 2 diabetes). The LDL-clearing activity of *LDLr* depends on the interaction between insulin and IR [1].

Insulin has been reported to stimulate *LDLr* expression, an IR-dependent signaling event [2]. The inactivation of IR and *LDLr* prevents the removal of extracellular LDL and promotes hyperproteinemia through intravascular LDL deposition. In insulin-deficient type 1 diabetes and insulin-resistant type 2 diabetes, IR inactivation leads to *LDLr* inactivation, which increases the severity of atherosclerotic complications due to the inability to remove vascular LDL.

Cholesterol in the brain builds up the membrane surfaces of large numbers of axon dendrites and synapses, including post-synaptic spines and pre-synaptic vesicles [3,4,5]. The brain comprises neurons and glial cells, which build up a large amount of membrane and occupy a high area and volume; thus, the cholesterol requirement is very high. Cholesterol metabolism is important in neurons and glial cells, which should cooperate for brain development and function, and *LDLr*, which interacts with IR, plays an important role in cholesterol synthesis and turnover regulation [6]. Cholesterol depletion in neurons impairs synaptic vesicle exocytosis, nerve activity, and neurotransmission, leading to dendritic spine and synaptic degeneration [7,8,9]. Furthermore, defects in cholesterol metabolism lead to structural and functional central nervous system (CNS) diseases such as Huntington’s, Alzheimer’s, and Parkinson’s diseases [10,11,12].

The hippocampus of an *LDLr*^−/−^ rodent model fed a high-cholesterol diet showed a higher neuroinflammatory response and impaired blood–brain-barrier (BBB) transport [13,14,15]. This affected the proliferation of hippocampal progenitors. Additionally, the neuroinflammatory process increases the production of ROS, which is detrimental to neurons [16]. Mitochondria are prone to oxidative damage, and inflammation can further contribute to mitochondrial dysfunction [17] and GSH-dependent antioxidant system damage. Several studies have reported an association between high dietary exposure to fat or cholesterol and oxidative stress in the rat and rat brain [18,19].

It is necessary to prove the correlation between diseases such as hypercholesterolemia, which are caused due to *LDLr* defects, with respect to brain metabolic physiology. Therefore, this review aims to provide a detailed overview of *LDLr*-defect-mediated metabolism disorders.

## 2. Cholesterol Regulation of *LDLr*

*LDLr* is a cell membrane glycoprotein that LDL, a cholesterol transporter, binds and internalizes. *LDLr* is a key receptor for maintaining cholesterol homeostasis by removing LDL through endocytosis, and is essential for lipoprotein and lipid metabolism. [20,21,22,23,24,25].

**Figure 1:**
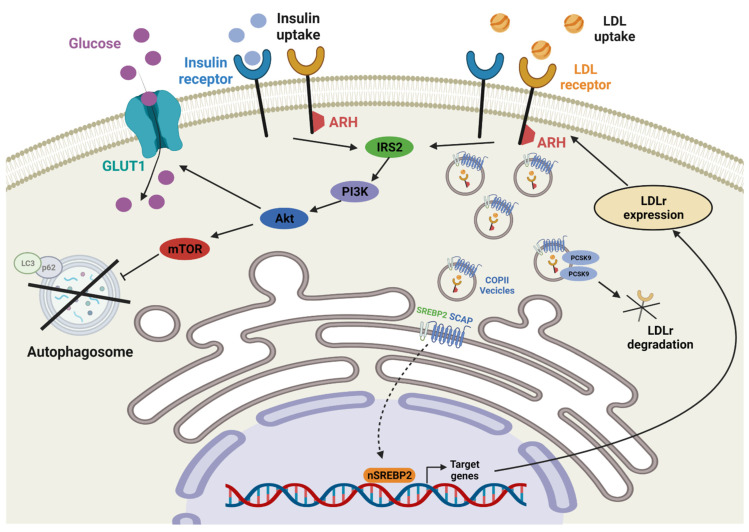
Interaction of low-density-lipoprotein receptor (*LDLr*) and insulin receptor (IR). The process of the expression of *LDLr* and endocytosed downstream molecules to regulate cholesterol homeostasis and the association of co-localized IR.

When cholesterol accumulates or decreases, the endoplasmic reticulum (ER) detects the level of membrane cholesterol and activates the cholesterol regulatory system (Figure 1), the sterol regulatory element-binding protein (*SREBP*) pathway, to maintain cholesterol homeostasis. When the intracellular cholesterol level is low, *SREBP* forms a complex with the polytopic membrane protein *SREBP* cleavage-activating protein (*Scap*) in the form of vesicles coated with coat protein complex II (COPII) in the ER, and the vesicles are then transported to the Golgi. Upon transporting the *Scap*/*SREBP* complex, *SREBP* is proteolytically degraded into an active fragment, which activates genes involved in cholesterol synthesis and absorption [26,27]. *SREBP*2 is activated by a reduction in intracellular cholesterol and induction of genes such as proprotein convertase subtilisin/kexin type 9 (*PCSK9*) and *LDLr*, leading to the endocytosis of 3-hydroxy-3-methylglutaryl coenzyme A reductase (*HMGCR*) LDL [27,28,29]. On the other hand, when the cholesterol level is high, the *Scap*/*SREBP* complex binds to Insig-1 or Insig-2, another polytopic membrane protein, and the coating of the *Scap*/*SREBP* complex is blocked by COPII and maintained in the ER. By preventing *SREBP* from moving to the Golgi [30,31,32], the transcription of target gene decreases cholesterol synthesis and absorption [28].

*LDLr* relies on *ARH*, a low-density-lipoprotein receptor adapter protein, for LDL internalization. *ARH* is mediated via S-nitrosylation by nitric oxide, and LDL is absorbed into the *LDLr*. In *ARH*^−/−^ cells, *LDLr* activity is inhibited due to *ARH* loss via *LDLr* endocytosis failure by the induction of *LDLr* redistribution into the plasma membrane [33,34,35,36,37]. *PCSK9* can degrade *LDLr* internalized in lysosomes and protect cells from excessive LDL uptake and cholesterol accumulation [38]. *HMGCR* activates the acetyl-CoA pathway, producing cholesterol as the final product [39].

## 3. Interaction of *LDLr* and IR

*LDLr*, a transmembrane glycoprotein, is involved in regulating blood cholesterol by binding and internalizing LDL containing cholesterol [40,41,42,43]. *LDLr* removes cholesterol-containing LDL particles [41,44], and IR maintains glycemic homeostasis [45,46,47]. On the other hand, although the exact mechanism is not completely clear, diabetes is associated with dyslipidemia [48]. Diabetes mellitus is characterized by metabolic abnormalities due to insufficient insulin production due to loss of beta cells (type 1 diabetes) or abnormalities in the insulin receptor protein itself (type 2 diabetes). Insulin is known to regulate several biological activities [49,50,51], resulting in increased glucose transport and the maintenance of adequate blood glucose levels [52].

*LDLr* and IR co-localization in organelles has been observed via electron microscopy. Increased insulin levels increase the LDL uptake of HepG2 cells via *LDLr* due to the disruption of the *LDLr*–IR co-localized complex; they further promote the internalization of extracellular LDL particles by directly regulating insulin-mediated *LDLr* function [53,54]. We have demonstrated the co-localization of *LDLr* and IR by reducing the expression of *LDLr* and IR-related proteins via *LDLr*-specific siRNA treatment [1].

In LDL exposure, autophagosome formation is suppressed via PI3K/Akt/mTOR activation, a key autophagy regulator in HUVECs, similar to the insulin pathway, and LC3 and p62 expression in the lipidized form of the autophagosome decreases. After LDL treatment, *LDLr* and IR expression increases in the cytosol rather than the membrane, indicating endocytosis from the cell membrane to cytosol, opposite to *GLUT1*’s translocation from the cell cytosol to membrane [55,56].

The impairment of IR function is associated with abnormal glucose metabolism leading to impaired glucose tolerance or diabetes, a condition of obesity and insulin resistance [57]. Insulin resistance is also associated with intravascular LDL accumulation due to poor clearance by functionally impaired *LDLr*. Insulin resistance results in decreased expression levels of insulin and IGF receptors, which promote neurite growth, synapse formation, and neuronal survival in the brains of Alzheimer’s patients [58]. The intraventricular injection of streptozotocin, which induces diabetes in rats, or the depletion of neuronal IR results in cognitive decline [59,60,61,62,63].

## 4. Cholesterol Metabolism in the Brain

Cholesterol is a structural component of the membrane that acts as a buffer for changes in the fluidity of cell lipid membranes and is involved in membrane-intrinsic proteins and cell signal transduction [64,65]. Despite these important functions, high cholesterol levels can be toxic to cells. An overload of total cellular cholesterol in the plasma membrane triggers its migration to the ER, resulting in the depletion of calcium stores, leading to cell death [66,67]. Additionally, a loss of membrane fluidity due to increased cholesterol levels can lead to dysfunction of the integral membrane protein and damage to the membrane domain, resulting in the disruption of signaling events [68].

Consequently, sterol-sensing proteins that regulate cholesterol homeostasis, sterol synthesis and degradation by regulatory mechanisms, and *LDLr* are also involved [69]. The brain is the most cholesterol-rich organ in the human body and contains 25% cholesterol [70,71]. Cholesterol homeostasis may influence neuroinflammatory expression for *PCSK9* and neuronal receptors. *PCSK9*, which can regulate the cholesterol receptor *LDLr* and apolipoprotein E (*ApoE*), maintains a certain concentration of cholesterol in the human cerebrospinal fluid under normal conditions [72,73]. Under increased BBB permeability in disease states, *PCSK9* crosses the BBB and induces *LDLr* degradation in the brain [74,75].

High levels of LDL-C have been observed in stroke patients with mutations in *LDLr*-related genes. Sequencing has been performed in stroke patients with *LDLr*, apolipoprotein B (*ApoB*), and *PCSK9* gene targets, and the familial hypercholesterolemia pathogenic gene may indicate atherosclerotic phenotypes such as increased carotid intima–media thickness and ischemic attacks [76,77,78,79].

## 5. BBB Breakdown

The BBB is formed and maintained by cerebral capillaries, pericytes surrounding the capillaries, and endothelial cells surrounding astrocyte ends that surround these two layers. The BBB, composed of adherens junctions (AJs) and tight junctions (TJs), selectively regulates the transport of molecules and cells in and out of the brain, thereby regulating the brain microenvironment [80,81]. It is composed of TJs comprising transmembrane proteins such as *occludin* and claudins and AJs comprising VE-cadherin, which plays an important role in maintaining brain endothelial junctions. The BBB maintains the homeostasis of neurovascular units, including blood vessels and nerve cells [82].

Many studies have demonstrated the potential for BBB disruption to alter brain regions and dissociation states, allowing neurotoxic plasma components, blood cells, and pathogens to enter the brain and cause neuroinflammation [83,84]. *LDLr*^−/−^ mice exposed to a high-cholesterol diet and accompanied by a decreased gene expression *of claudin-5* and *occludin* and increased BBB permeability to stimulate neuroinflammation and cognition are more susceptible to the disorder, and BBB disruption is associated with brain changes due to hypercholesterolemia [75]. In *LDLr*^−/−^ mice fed a high-cholesterol diet, aquaporin-4 (AQP-4) expression increases [85,86], *occludin* and *claudin-5* gene expression decreases, BBB permeability increases, and GFAP-derived neuroinflammation increases in the hippocampus [75].

## 6. Neuroinflammation

**Figure 2:**
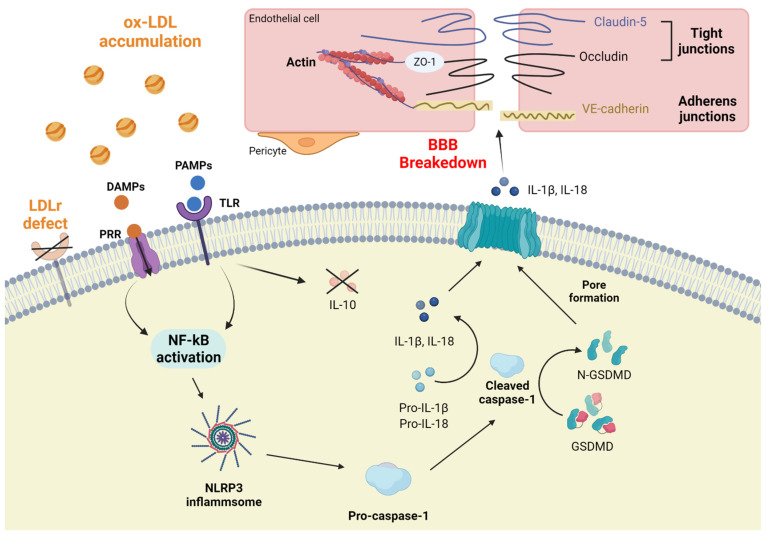
Blood–brain-barrier (BBB) breakdown due to inflammation. BBB breakdown process induced by NLRP3-mediated inflammation.

*LDLr* is important for regulating LDL homeostasis. LDL is oxidized, producing oxLDL. Although LDL-C levels decrease in the sera of patients with rheumatoid arthritis, a chronic inflammatory disease, high levels of oxLDL [87] produce cytokines such as TNF-a and IL-6 [88,89]. After cerebral I/R injury, the downregulation of *LDLr* expression in neurons, astrocytes, and oligodendrocytes [90] leads to neurological deficits, infarction, and edema in the CNS. Additionally, *LDLr* KO activates pattern recognition receptors (PRRs) present in innate immune cells with damage-associated molecular patterns (DAMPs) generated in damaged cells or tissues (Figure 2) [91], and in the middle cerebral artery occlusion (MCAO) model under ischemic conditions. Additionally, LDL accumulation was proven to be inflammatory by inducing TLR activation via pathogen-associated molecular patterns (PAMPs) [92,93], and oxLDL, acting as a DAMP and PAMP, forms the *NLRP3* inflammasome complex [94,95]. The complex induces gasdermin D (GSDMD)-mediated lytic apoptosis (pyroptosis) by increasing the levels of active caspase-1. Pyroptosis, a programmed cell death process, is mediated by a pore formed by the binding of GSDMD N-terminal fragments cleaved by active caspase-1 to the plasma membrane, inducing the release of inflammatory cytokines such as IL-1β and IL-18, leading to neuronal pyroptosis [96,97,98,99,100]. Additionally, interleukin-10 (IL-10), an anti-inflammatory cytokine, inhibits the activity of inflammatory cytokines by inhibiting *TLR* induction [93].

The blockade of NLRP3 delays neuronal pyroptosis in *LDLr*^−/−^ mice and cultured *LDLr*^−/−^ neurons after experimental stroke [91]. Oxidative stress and mitochondrial DNA (mtDNA) damage have been observed in atherosclerotic plaques in *LDLr*^−/−^ mice, and the number of atherosclerotic plaques increase due to an additional deficiency of Ogg1 promoting NLRP3 inflammasome activation in *LDLr*^−/−^ mice. The mitochondria and mtDNA, which are sites for reactive oxygen species (ROS) production, are vulnerable to oxidative stress due to a lack of protective histones. *Ogg1*, a glycosylase responsible for the cleavage of 7,8-dihydro-8-oxo-2’-deoxyguanosine, a byproduct of ROS, and AMPK, an upstream regulator, are targets of miR-33, a proatherogenic microRNA. As a result, the levels of Ogg1 and AMPK decrease in atherosclerotic plaques [101].

*LDLr* overexpression is associated with inflammation relief. Lipopolysaccharides (LPS), which are made up of lipids and polysaccharides, can cause inflammation. They have the highest binding affinity for high-density lipoprotein (HDL), and HDL-binding LPS is redistributed to LDL and VLDL [102]. Pathogen lipids such as LPS are integrated and transduced into lipoprotein particles such as HDL, LDL, and VLDL, triggering an immune-inflammatory response through Toll-like receptors (TLRs). TLRs are mammalian PRRs that recognize structural pathogen-associated molecular patterns shared by microorganisms in innate immunity. The inhibition of *PCSK9*, which promotes the degradation of *LDLr* lysosomes, increases the ability of *LDLr* to remove pathogenic lipids, thereby reducing the inflammatory response [103,104].

## 7. Interaction between ER Stress and Mitochondria

*LDLr* deficiency can lead to cognitive impairment due to interactions between ER stress and mitochondria. In particular, the brain is vulnerable to oxidative damage and apoptosis induction by abnormal calcium and ATP levels due to its high energy metabolism rate, high oxygen consumption, and high ratio of polyunsaturated fatty acids [105,106,107,108,109,110]. Oxidative stress is attracting attention as a cause of several neurodegenerative diseases [111,112,113].

**Figure 3:**
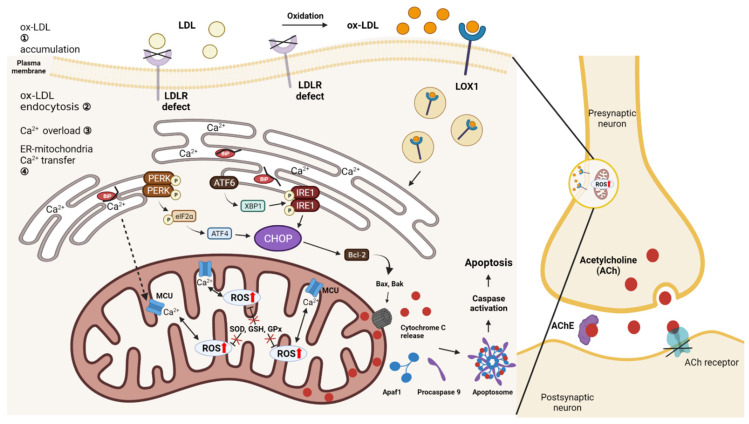
Interaction between ER stress and mitochondria. The process by which ER stress and mitochondria interact due to oxLDL accumulation due to *LDLr* deficiency affects cognitive impairment.

LDL is oxidized to oxLDL, which is taken up by lectin-like oxidized low-density-lipoprotein-1 receptors (Figure 3). OxLDL increases lectin-like oxidized LDL receptor-1 (*LOX-1*) expression in macrophages, leading to macrophage migration and foam cell differentiation, leading to deposition into endothelial cells [114,115]. OxLDL uptake induces Ca^2+^ overload, which further induces mitochondrial dysfunction, leading to cytochrome c release, the apoptosis of endothelial cells, and the suppression of antioxidant activity, generating ROS that can activate NF-κB [116,117,118,119,120,121,122].

Abnormally folded proteins in the ER lumen are increased in atherosclerosis resulting from cholesterol accumulation. The three major ER-stress sensor proteins of the UPR that remove these abnormally folded proteins are inositol-requiring enzyme 1 (IRE1), activating transcription factor 6 (ATF6) and protein kinase RNA-like ER kinase (PERK). In the steady state, the three sensor proteins remain inactive due to their binding to the ER chaperone, glucose response protein-78 (BiP/GRP78). Stress releases BiP from the ER sensor and induces the phosphorylation and dimerization of IRE1 and PERK in cancers, whereby active PERK promotes the phosphorylation of eukaryotic initiation factor 2α (eIF2α) and inhibits protein translation by the activation of activating transcription factor 4 (ATF4) [123,124], and X-box binding protein 1 (XBP1) expression is achieved through ATF6 activation [125]. However, prolonged cellular stress induces CHOP expression, and a CHOP-mediated imbalance in the Bcl-2 family activates proapoptotic proteins in the mitochondrial membrane, inducing the release of cytochrome c, leading to subsequent mitochondrion-dependent apoptosis [126,127].

In pathological conditions such as ischemia and reperfusion, the depolarization of the mitochondrial membrane potential increases, causing Ca^2+^ overload in the outer membrane and Ca^2+^ conduction through the calcium uniporter (MCU), which is locally present in the inner mitochondrial membrane. Increased Ca^2+^ is produced due to a bidirectional interaction with ROS [128,129,130,131]. The mitochondrial outer membrane channel is formed by the opening of the mitochondrial permeability transition pore (mPTP), which induces mitochondrial membrane permeability and the insertion of proapoptotic BH3-domain-containing proteins such as Bcl-2-associated X protein (Bax). In addition, due to the depletion of cardiolipin, a lipid constituting the inner membrane of mitochondria, cytochrome c is released from the inner membrane of mitochondria into the cytoplasm [132,133]. Cytochrome c forms an apoptosome and induces apoptosis by forming a complex with apoptosis-protease activating factor 1, which is required for the proteolysis of caspase-9 and caspase-3 [134,135]. In the mitochondria, ROS are formed as a byproduct of oxidative phosphorylation that induces ATP production. Imbalances in ROS, ATP, and Ca^2+^ that appear in mitochondrial dysfunction increase the expression of antioxidant enzymes, such as superoxide dismutase (SOD), glutathione (GSH), and glutathione peroxidase (GPx), and the Bcl-2-mediated apoptosis mechanism [136]. Furthermore, oxidative stress also affects endothelial cells.

Hypercholesterolemia-induced *LDLr*^−/−^ mice have shown a decreased activity of mitochondrial complex I and II in the cerebral cortex; decreased GSH; an approximately 40% increase in complexes formed via thiobarbituric acid’s reaction with malondialdehyde (MDA), a product of lipid peroxidation; and enzymes related to peroxide removal. An imbalance in phosphorus GPx/glutathione reductase activity results in mitochondrial dysfunction and oxidative stress, leading to cognitive impairment [137,138]. In 14-month-old *LDLr*^−/−^ mice, antioxidant imbalance and glutathione metabolism increase due to brain oxidative stress because of lipid peroxidation, and the acetylcholinesterase activity, which degrades the neurotransmitter acetylcholine, also increases [139]. The memory deficit observed in *LDLr*^−/−^ mice is not related to Aβ-level changes in the prefrontal cortex and hippocampus; however, Bcl-2 expression and caspase-3 activation decrease, while Bax expression increases.

## 8. *ApoE*

**Figure 4:**
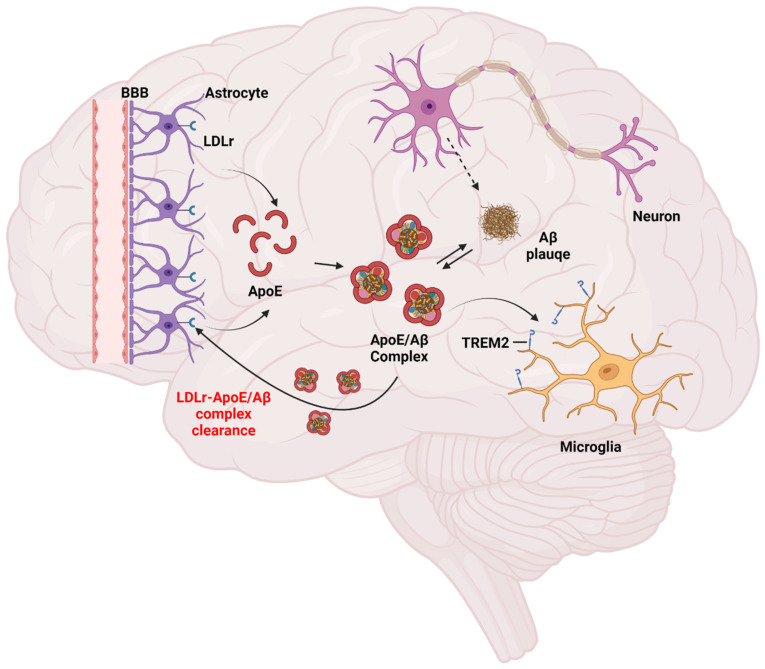
The process of the clearance of *ApoE*/Aβ complexes by *LDLr*.

Low-density-lipoprotein receptor (*LDLR*) and apolipoprotein E (*ApoE*) are responsible for the transport of cholesterol-rich lipoproteins (Figure 4). Deficiencies in *LDLR* and *ApoE* are associated with increased plasma total cholesterol and, consequently, a higher risk of hypercholesterolemia, atherosclerosis, and coronary artery disease [140,141,142]. *ApoE* is mainly produced by astrocytes and is a protein that plays a role in lipid transport in the CNS [143]. Unlike the *LDLR*-binding molecule *ApoB*100, small fat-soluble molecules such as *ApoE* can cross the BBB-forming membrane and affect BBB stability [144]. *ApoE* was found to co-localize with amyloid plaque [145,146]. *ApoE* exists in three isoforms: *ApoE*2, *ApoE*3, and *ApoE*4. *ApoE*4 induces brain damage as traumatic brain injury (TBI) [147], Alzheimer’s disease [148], and conditions leading to impaired cognition [149] do.

*LDLR* is an important receptor for *ApoE* in the central nervous system. [10.1016/j.nbd.2004.01.015] (accessed on 26 July 2022) Amyloid beta (Aβ), known to cause Alzheimer’s disease, is hypothesized to accumulate in the brain as plaques and is regulated by receptors for *ApoE* [150,151,152]. *LDLr* is expressed in astrocytes and induces the uptake of *ApoE* and Aβ [153]. It prevents Aβ deposition by reducing *ApoE* levels in *LDLr*-overexpressing mice [154]. *LDLr* is associated with the major central nervous system *ApoE* receptor regulating amyloid deposition in a distinct mouse model of β-amyloidosis. The acute intraventricular injection of aggregated Aβ(1–40) peptides was used to show increased susceptibility to Aβ-induced neurotoxicity, with intrahippocampal oxidative stress, neuroinflammation, nerve membrane damage, memory deficits, and increased blood–brain-barrier permeability [155].

*ApoE* also affects the immune response, and in mice, *ApoE* KO increased the expression of Toll-like receptor 4 (*TLR4*) and *LOX-1*, suggesting the formation of foam cells and promoting the onset of arteriosclerosis [156]. Additionally, *ApoE* promotes phagocytosis by binding to triggering receptor expressed on myeloid cells 2 (TREM2) in microglia [157]. *LDLr*^−/−^ mice exhibit reduced numbers of synaptophysin-immunoreactive presynaptic boutons in hippocampus CA1 compared to *LDLr*^+/+^ mice, resulting in hippocampus-dependent memory function impairment [0].

## 9. Conclusions

Our analysis revealed that *LDLr* defects might lead to IR interactions, BBB breakdown, neuroinflammatory responses, interactions between ER stress and mitochondria, and hypoxia. There are various cells in the brain that make up a complex cellular network. Each cell exhibits a lipid membrane structure as an essential element for cell maintenance and signal transduction, and lipids are then delivered by LDL, which is regulated by *LDLr*.

However, studies on *LDLr* defects have mainly been conducted in the liver, which is the main organ for lipid synthesis; thus, there is a lack of studies on changes in hormones such as cortisol, insulin, and leptin secreted into the brain signaling system and the mechanism of *LDLr* regulation specifically for brain cells.

In conclusion, this review summarizes the relationship between *LDLr* defects and brain metabolism. Further detailed studies are required to elucidate the mechanisms involved in *LDLr*.

## Data Availability

Not applicable.
